# Developing trauma-informed university supports for refugee background students in Australia: Refocusing through an ethics of care lens

**DOI:** 10.1007/s13384-023-00625-9

**Published:** 2023-04-28

**Authors:** Sally Baker, Loshini Naidoo

**Affiliations:** 1 School of Education, University of New South Wales, Kensington, Australia; 2 School of Education, Western Sydney University, Penrith, Australia

**Keywords:** Students from refugee backgrounds, University supports, Higher education, Trauma informed, Ethics of care

## Abstract

The growing literature on access, participation, and success of refugees entering higher education has illustrated the myriad challenges that this cohort faces. Much of this research has rightly focused on the student perspective, exploring the barriers and challenges that impede entry, engagement, and achievement. Relatedly, there is growing attention to the need for trauma-informed support, particularly following the impacts of COVID on learning. This article takes these challenges as a departure point to adjust the gaze on universities and ask what needs to be considered and implemented in order to develop better student supports. We use Tronto’s (2013) notion of ethics of care—examining issues of attentiveness (caring about), responsibility (caring for), competence (caregiving), responsiveness (care receiving), and trust (caring with)—to carefully probe how universities can develop trauma-informed supports that are more caring and nuanced, not only for students from refugee backgrounds but for all students.

## Introduction

As the numbers of refugees and displaced persons worldwide continues to rise, it is inevitable that the number of students from refugee backgrounds (SfRBs)^1^ seeking access to education will increase. In this article, we focus on the case of Australia as a settlement country,^2^ which has a relatively generous humanitarian migration program (per capita)^3^. However, we know little about SfRB access and retention in Australian universities because of a lack of data gathered on this cohort. Molla’s (2022) analysis of Commonwealth Department of Education data regarding refugee african youth (RAY) participation in higher education worryingly suggests that 83% paused or withdrew from their studies between 2001 and 2017. This high level of attrition suggests that while SfRB are able to access university study, they are not necessarily thriving.

Therefore, this clear imperative demands that we rethink how we support the educational opportunities and outcomes of SfRBs. The scholarly literature attests to the significance of accessing forms of tertiary education for SfRB (Naidoo, 2015; Stevenson & Baker, 2018) because education offers hope and opportunity, often disrupting the bleak daily experience of waiting for settlement or improving the radical disruption of early settlement. However, although education is known to be important, there is concern that the educational systems and structures in settlement contexts such as Australia lack the flexibility needed to respond to the support needs of SfRBs (for example, Baker et al., 2018; Naidoo, 2015), especially those who experience the impacts of trauma. There is an undeniable need for educational institutions to create generative spaces for SfRBs by reconceptualising learning as a holistic process that extends beyond classroom walls, recognising the impacts that trauma causes, and building on the assets these students bring to learning (Naidoo, 2015; Wilson, 2022).

In this article, we will focus on the challenges of supporting access, participation, and success for SfRBs in the higher education sector, although we fully recognise that all parts of the education system (early childhood, schooling, vocational education and training, adult/ community education and higher education) are an interconnected ecology. This article explores how universities might reframe support for people from refugee backgrounds using a trauma-informed reading of Joan Tronto’s (1993, 2013) ethics of care.

### Students from refugee backgrounds and higher education in settlement contexts

Access to higher education in settlement countries such as Australia is particularly challenging for students whose educational trajectories have been marred by periods of disruption due to forced migration, and where cultural and linguistic diversity can be viewed as limitations rather than strengths by institutions. SfRBs are a ‘super-disadvantaged’ cohort (Lambrechts, 2020) because of the constellation of challenges that they are likely to face due to their pre- and post-forced migration experiences. A growing body of scholarship has explored the barriers to access and participation, including information barriers (Bajwa et al., 2017), language development (Baker et al., 2018), transition (Baker & Irwin, 2021; Naidoo et al., 2018), issues of institutional support (Mangan & Winter, 2017), different understandings of time (Baker et al., 2020), and issues related to online teaching (Halkic & Arnold, 2020). While this article focuses on the Australian context, the issues described here are pertinent to other settlement contexts, with similar challenges noted in countries such as the UK (Lambrechts, 2020; Mangan & Winter, 2017), Canada (Bajwa et al., 2017), the US (McWilliams & Bonet, 2016), Germany (Streitweiser & Brück, 2018) and Spain (Marcu, 2018).

While many public Australian universities have access pathways and some financial supports for SfRBs, there is little in place to support this cohort with participating and succeeding in their studies, and limited support for transitioning out of their studies into employment or further study (Baker et al., 2021). Where supports do exist, there is significant variability in terms of what and how this operates across the Australian higher education sector. While some universities have multiple scholarships, offer mentoring, and employ people to specifically support SfRBs, other universities do very little. The reasons for this disparity are not documented, but the varying levels of activity likely corresponds with SfRB enrolment numbers and geographic locations of universities in high-density migration zones, some universities are doing more than others to support the needs of this marginalised group.

In Australia, increasing scholarly and practitioner interest in improving the educational opportunities and outcomes for SfRBs arguably emerges from a lack of national policy and funding attention for this cohort. Their invisibility in equity policy means that explicit institutional support for SfRBs may not exist because of a lack of mandate for universities to target this cohort. A further challenge is contributed by the lack of available data on the SfRB cohort, with universities not required to capture information on this group because they are not a nominated ‘equity’ cohort (Perales et al., 2021). Moreover, the inconsistency in responses across Australia exemplifies how universities interpret how equity funding should be spent—flexibly to respond to the needs of their student cohort, or narrowly to fit in with the policy.

Overall, exploring higher education through the lens of SfRBs helps us to resist the assumptions that universities make about who our students are, what they can do, what they bring, what they need, and what they desire (Stevenson & Baker, 2018). By thinking about how SfRBs access, participate in and transition out of higher education, we can see that the sector, in Australia at least, fails in its duty of care because it continues to employ a hegemonic lens to design its systems and policies with ‘traditional’ students^4^ in mind. When viewing higher education experiences from the eyes of a refugee student, we are forced to unpack paternalistic assumptions about our students’ prior educational experiences, about cultural and linguistic diversity, about ethnicity and religious background, and about experiences of trauma, displacement, and conflict. However, describing the challenges for this cohort—whether at the policy, structural, systemic, institutional, or individual levels—will only take our advocacy so far. To be able to effect meaningful and sustainable change, we need to think about where the opportunities lie for shifting the assumptions that frame policy, funding, and practice. One way to do this is to consider how trauma impacts on education, and what this means for the ways that universities design and provide support.

## Trauma and learning

As we learn more about how trauma impacts survivors of distressing events, we are also developing more nuanced understandings of the consequences of trauma on learning. Trauma as defined by the Substance Abuse and Mental Health Services Administration (SAMHSA), results from experiences that have ‘lasting adverse effects on the individual’s functioning and mental, physical, social, emotional, or spiritual wellbeing’ (2014, p. 7). We know that trauma manifests differently in individuals, and it is, therefore, important not to essentialise trauma. However, decades of research tell us that there are patterns in how the ‘traces’ of trauma are experienced in the brain and in the body (Van Der Kolk, 2014), including hypervigilance, depression, antisocial violence, self-violence (Berger & Martin, 2020), and can endure over a lifetime. We also know that many people experience trauma, to differing degrees, and they can respond differently according to different stressors and conditions. Moreover, as Van Der Kolk reminds us, ‘Trauma affects not only those who are directly exposed to it, but also those around them’ (p.1). This offers an important reminder to why we need to consider trauma in our interactions with others, particularly in teaching and learning contexts, although we recognise that not all SfRB experience trauma, and if they do, their responses are likely to be divergent.

### Trauma and higher education learning

Students who are impacted by trauma may feel disconnected from the support of others and have no shared connection or sense of belonging to the university community. As Wilson (2022) shares, to better support these students, the university environment needs to be nurturing and relational, while simultaneously acknowledging students’ capabilities and capacities to improve their own engagement. Similarly, Stephens (2020) links trauma-informed pedagogy and care, explaining that a trauma-informed approach necessitates a shift from ‘confrontation to care’ in higher education. This requires educators to explore how mis/understandings of trauma impact their ‘pedagogical effectiveness and ability to nurture the best learning in their students’ (p. 1).

In considering trauma, Substance Abuse and Mental Health Services Administration (SAMHSA) identify four Rs for becoming trauma-informed that can be applied to educational systems and supports:

1. *‘[R]ealize* the widespread impact of trauma and understand potential paths for recovery;
2. *recognise* the signs and symptoms of trauma in students, staff, and others involved with the system;
3. *respond* by fully integrating knowledge about trauma into policies, procedures, and practices; and
4. seek to actively *resist* re-traumatization’.

(SAMHSA, 2014, p. 9).

In this article, we draw on these four Rs in dialogue with Tronto’s (1993, 2013) five elements of an ethics of care—attentiveness, responsibility, competence, responsiveness, and trust—to probe universities’ responses to the needs of SfRBs and consider ways for how we might change business as usual.

## Ethics of care

An ethics of care foregrounds relationality and situatedness in considering ethical issues and moral judgements. The notion of an ethics of care has its roots in the western feminist-critical tradition, offering an alternative to the more autonomous ideas behind ethics of justice. As a political scientist, Tronto’s (1993, 2013) work explores the politics of care, noting how individualisation and the dominance of economics serves to invisibilised caring relations, often in highly gendered ways. She notes that care involves a moral judgement and one that involves taking the standpoint from the view of the other, from their lived experiences and from the particular needs of others. This notion of care goes beyond just taking an interest in another person, suggesting that it will lead to action.

Tronto’s work on the politics of care is useful for exploring how macro (government) and meso (institutional) domains create a political economy that devalues care over production and profit, and denies the intricate caring relationships we are all involved in. Foregrounding caring relationships and commitments to other, Tronto’s (1993, 2013) five elements of an ethics of care—*attentiveness, responsibility, competence, responsiveness,* and *trust*—offer a useful framing for our discussion of how universities can respond to the needs of SfRBs. These include:

1. Attentiveness (caring *about*): noticing unmet needs, suspending one’s own judgements and being able to see the world from the perspective of the one in need.
2. Responsibility (caring *for*): taking on the burden of responding to this need.
3. Competence (care *giving*): being competent to care, which is always both a technical and a moral and political issue.
4. Responsiveness (care *receiving*): listening to the response of the person/group that was cared for, sometimes resulting in new unmet needs.
5. Solidarity (caring *with*): taking collective responsibility, to think of citizens as both receivers and givers of care, and to think seriously about the nature of caring needs in society.

While this idea comes from a western standpoint, in the decentring a focus on individuals, and pushing back against what Tronto calls ‘privileged irresponsibility’, ethics of care shares a concern about inter-relatedness, with subaltern and Indigenous ideas about caring, such as *ubuntu* and aboriginal notions of kinship care. Ubuntu and kinship ethics of care acknowledge our interconnectedness and shared rhythms, thus, resisting a denial of a person’s right to belong to humanity (Waghid, 2019, 90). Thus, these non-western relational ethics of care offer opportunities to prevent and resolve the human conflict/injury that western/ individualised modes of care can create; instead they help work towards equality, compassion, and reconciliation Moreover, while we acknowledge the term ‘needs’ used throughout this article has ‘saviour’ overtones, particularly when used in the institutional context of higher education, we use this to refer to the specific support requirements that students may have (self-identified and probable, based on cohort-specific knowledge). We certainly do not intend to reify or presuppose ‘needs’.

In this article, we seek to align these five ethical components of care with SAMHSA’s four Rs of trauma responsiveness. We see connections between *attentiveness, responsibility and competence* and **realising** and **recognising**, *responsiveness* and **responding,** and *solidarity* and **resisting**.

### A note on our positionality

We are academics with a deep commitment to contributing to more socially just university systems through our research, teaching, and advocacy with refugees and asylum seekers. We believe that our scholarship and our positions as educators in the academy can serve those goals and ‘make visible our own critical positioning within the structure of power’ (McDowell, 1992, 413). We are not trying to legitimise our positions here; rather we hope to show the complementarity of our academic and research backgrounds to the discussion in the paper, and illustrate the porous boundaries between emotions—emphasising strength, commitment, and care—and our work with marginalised groups.

### Ethics of care in higher education

In the higher education sector, a focus on care sits at odds with the neoliberal logics that have created the conditions for ‘undercaring’ institutions (Bosanquet, 2017). As Warin (2014) notes, an ethics of care is necessary given how the ‘priority for economic competition, as a driver of education, [has been] accompanied by a reduction in public and visible forms of care and welfare’ (p. 100). A key to the success of the neoliberal higher education project is the dominance of assumptions of autonomy, which belies a lack of willingness to recognise that we are all vulnerable and all involved in caring circles, which results in care being undervalued and invisibilised.

Applying an ethics of care lens to higher education—thus, foregrounding relationships, needs and responses, and the imperative to care/ be kind/ show compassion—can help educators to challenge ‘post-truth’ notions that undermine and ignore the vital role that care plays when building substantial, professional, and caring relationships between people working in universities, SfRBs and community members. As Bozalek et al. (2014) note, there is a clear political dimension to applying a care gaze to complex contexts such as higher education, with the critique exposing hegemonic and unforgiving assumptions and mechanisms that—perhaps inadvertently—disadvantage particular groups, and ‘provides a way of establishing where imbalances between the elements may be impacting on how well care is practiced’ (p. 457). In the case of higher education, these disadvantaged groups include female staff members, who are more likely to be the care foot-soldiers who ‘do’ care (Grummell et al., 2009), and ‘non-traditional’ students, including those outside of formal equity categories, such as SfRBs, student-parents, and mature-age students.

## Exploring the potential for trauma-informed higher education supports

Tronto’s five elements of an ethics of care focus on knowledge, attitudes and practices required by educators to be exemplary/quality teachers for refugee background students. By responding to each element in turn through the lens of SfRBs, we critically examine what needs to be considered to develop better structures and supports according to an ethics of care.

### Attentiveness (caring about)

*Caring about* involves assuming the position of the ‘other’, so as to recognise the need for care and caring needs (Tronto, 1993) and extends to care-about-ideas (Barnacle & Dall’Alba, 2017). The quality of *attentiveness* is what keeps educators concerned about the lives of students and avoids them overlooking the fact that those lives are of value in their own right. It means educators achieving attentiveness through dialogue with students, acknowledging that each of us is differently located—economically, socially, linguistically, culturally—with different life histories, experiences, expectations, wealth, and frames of reference. *Caring about* also overlaps with responsibility, in terms of not assuming needs and making m/paternalistic decisions that erase autonomy and agency to self-advocate or decide for oneself what the best course of caring action should be. This requires training for educators and students to be able to **realise** and **recognise** (SAMHSA’s trauma responses) in ways that activate specialist supports. The recognition of care for SfRBs is about asking students what they need so as to identify the uniqueness of their life experiences and journeys, and to try and understand those places outside the realm of others’ experiences. Through the development of opportunities to ‘listen with intent’ (Matthews & Sunderland, 2017), universities will be better placed to develop a suite of supports that can **respond** to bespoke needs of different student cohorts. This must include consideration of how the trauma of forced migration impacts on SfRBs’ mental health, wellbeing, and learning (Wilson, 2022).

Similarly, universities should also recognise that students (and staff) cannot always make agentic decisions about their own needs because they do not have access to a full suite of information about the systemic context, the structural arrangements, or have expertise on specific cohorts. In such cases, the attentiveness should be shared with experts (such as trauma specialists, language teachers, settlement services, and community members in the case of SfRBs) to ensure that meaningful contribution can be brought into dialogue with those seeking support.

### Responsibility (caring for)

Once the conditions for listening, attending and caring-about are established, responsibility must be taken. Caregivers need to take into consideration how the care receivers feel about the care that is given. Tronto (1993) sees responsibility as ‘embedded in a set of implicit cultural practices, rather than a set of formal rules or series of promises’ (p. 132), with caring for encompassing not only person-person care, but also care about the environment, non-human beings, artefacts, and knowledge. Caring for also requires caregivers and receivers to **recognise **the signs and symptoms of trauma and then assume responsibility for improvement.

In the higher education context, Barnacle and Dall’Alba (2017) define care for students as involving being responsive to their interests and capacities. They write, ‘Importantly, it also acknowledges multiple learning trajectories in promoting students’ being and becoming’ (p. 7). This recognition of multiplicity and ambiguity requires reflexivity (on the part of caregivers), so universities are supportive of care without domination. Moreover, *caring for* also includes ensuring that curriculum knowledge, teaching practices and assessment strategies are trauma informed and equitable; this means moderating existing practice with diversity and inclusion considerations. This could include examples such as adapting content to increase the diversity of voices and perspectives included, moderating colloquial language use to avoid being exclusive, and designing assignments that can accommodate students’ needs, or creating culturally safe conditions for assessment to take place.

With SfRBs in particular, *caring for* can include ensuring that learning materials are accessible in terms of language, that content does not trigger past trauma, the development of workarounds to meet students where they are (such as the idea of plurilingual course glossaries to minimise the impact of the language load; see Ollerhead & Baker, 2019). *Caring for* requires the caregiver to step outside of their own frame of reference—to displace one’s motivation according to Nel Noddings (1984)—to see from the perspective of another and adjust appropriately. In the teacher–student interaction, Noddings (1984) argues that this involves ‘mak[ing] the[ir] problem my own, receive it intellectually, immerse myself in it; I must also bring the student having difficulty into proximity, receive such students personally’ (p. 113). However, this component of caring comes with a warning. ‘Caring for’ can be understood as a burden (Swartz, Gachago & Belford, 2018), with responsibility bringing with it an emotional weight and vulnerability for the caregiver. With burnout a perennial concern in higher education (Hegney et al., 2021), taking responsibility and caring for students can be more than an individual is able to carry.

### Competence (caregiving)

Once the needs have been established, and responsibility for meeting care needs has been assumed, the provision of care depends on the competence of the caregiver in terms of their ability to do so. Tronto (1993) reminds us that caring competently depends upon adequate resources: ‘on material goods, on time and on skills’ (p. 110), the first two of which are in short supply in contemporary academia.

A key part of caregiving, especially for educators in professional disciplines that involve a requirement of uneven and high stakes interaction between people, such as nursing, social work and teaching, is to model care. As Barnacle and Dall’Alba (2017) note in their essay, teachers have a responsibility to show students ‘how to care’, ‘not only in terms of promoting passion for ideas and objects, but also through students caring about each other in their interactions’ (p. 8). This idea has strong connections to the SAMHSA ‘R’ of **responding**—to do so, educators and students need to be aware of what supports are offered, and advocate for those that are needed but not available. This is a form of response/ive caregiving. Through modelling caregiving, this helps to move discussions of care beyond narrow conceptions of care as ‘wellbeing’—an important consideration but a seductively limiting discourse that serves to (largely) push responsibility for care onto the individual. The premise of this argument is simple: when students feel cared for and that they have been given care, they will be more likely to recognise this as a reciprocal relationship, and therefore, more likely to offer care to others. While we acknowledge that caregiving is rarely simple, highlighting the ways that we are all involved in caring relations is a core dimension in Tronto’s (2013) argument for creating caring democracies, and in trauma-informed support (Wilson, 2022).

In higher education, the resource deficiencies created by years of neoliberal austerity and recent pandemic-related shortfalls in funding have created sub-optimal conditions for caregiving. Blackmore (2020) scathingly outlines this as,

*…the carelessness of universities towards their employees has been magnified by university management responses to COVID-19. …. Policies encouraging ‘flexibility’ have, over decades, favoured the university with 65% of academics now on contract or casual terms.*

(p. 1334)

The impacts of COVID have highlighted the tightness in the system, where time and personnel resources have long been in short supply. Looking at higher education through the lens of SfRBs highlights the ways that the institution can be felt as uncaring, with endemic funding cuts, staff reviews and centralisation leading to high numbers of redundancies and reduced services. For a group of students who largely prefer people-rich rather than online supports and prefer trusted networks to formal institutional representatives (Baker et al., 2018), the reduction of staff can have significant consequences for their engagement with their studies. Certainly, the impacts of COVID have significantly and negatively impacted on SfRBs and the caregiving that they may have ordinarily received (Mupenzi et al., 2020). The pandemic has paradoxically highlighted both caregiving needs of all students, and the lack of proactive caregiving offered by universities. On a more positive note, it has also highlighted how universities can respond quickly when needed, as seen in the rapid shift to online learning.

### Responsiveness (care receiving)

The counter component of caregiving is care receiving, which assumes recognition that the receiver of care will respond to the care received, requiring a measure of whether our care (both caregivers and care receivers) has been effective. We should not rely solely on the market to determine the effectiveness of care, as it will disproportionately (only) measure the economics of the interaction, rather than the effectiveness of the relational, social, and emotional components of care. As Fisher and Tronto (1990) note, when care receiving is dictated by market principles, the care-receiver is often marginalised, ‘Because bureaucratic caring grows out of a political process that precludes control by care-receivers, much bureaucratic caring is fragmented and inadequate’ (p. 49). Moreover, recognition needs to extend beyond the assumption of a caregiver–receiver dyad; rather, we must remember that we are all receivers of care, even if not actively in the moment. As Tronto (2013) argues, this recognition of shared caregiving and receiving helps to disrupt ‘the dyadic model of care [which] serves to heighten our sense of ‘dis-ease’ and discomfort about asymmetry of care’ (p. 152).

Care receiving also extends to self-care and reflexivity, particularly in the educational encounter. As Noddings (1984) wrote,

*…teaching involves a meeting of one-caring and cared for… To teach involves a giving of self and a receiving of other… I must explain, question, doubt, explore, revise, discover, err and correct, but I must also receive, reflect and act.* (p. 113).

To do so requires us to build on the earlier dimensions (caring about, for, giving) and seek confirmation that our caregiving has been recognised/ that the care we need has been given. Feedback on this can measure the effectiveness of the care. If the care is not recognised/responded to, the caring relationship is put at risk and the interest in caring/being cared for may be damaged.

Inconsistent care, or ‘undercare’ (Bosanquet, 2017) is particularly unsafe for SfRBs, whose existence is likely influenced by traumatic past experiences. If a student feels disconnected, unsupported, uncared for, they are likely to drop out. Similarly, but less importantly, if an institution offers care (for example, in the guise of a targeted support program to help SfRBs develop their academic capacities), but the care is not recognised as such (students do not attend the program), the institution may withdraw their ‘care’. In such cases, universities (as the dominant partner in the caring relationship) should ensure that they cared sufficiently, drawing on trauma-informed practices (for example: was the program informed by students’ lived experience; were students asked what support/ resources they needed; did the university communicate adequately regarding the level of care available?). Attrition and withdrawal of resources can have significant implications for both parties, so programs need robust evaluation measures, with supports consistently refined to ensure the caregiver–receiver dynamic is sustained.

### Trust and solidarity (caring with)

The final dimension of care involves taking collective responsibility to think as both receivers and givers of care, and to seriously consider how we can craft a caring democracy. As Tronto (2013) writes, ‘Caring with’ requires that citizens care enough about caring—both in their own lives and in the lives of their fellow citizens—to accept that they bear the political burden of caring for their future’ (p. xi)—not just in economic terms, but also in terms of freedom, equality, and justice. In the higher education context, Vásquez Vedera (2019) argues these are foundations for collegiality and knowledge making. This layered definition is also evoked by Walker and Gleaves (2016), who argue that care can be emancipatory if it holds a ‘meaning and status of care as a mechanism to effect change, not just in pedagogic, but also social terms within education more generally’ (p. 67). Such political possibilities are concealed, however, with current conceptions of care as primarily pastoral (in terms of supporting students) and hived off as the responsibility of separate units, rather than a unifying and fundamental component of all parts of the university (Mariskind, 2014).

Advocacy for enhancing educational opportunities and outcomes for—and **resisting** (SAMHSA ‘R’) the retraumatisation of—SfRBs has a long history in higher education, with the Refugee Education Special Interest Group^5^ and Academics for Refugees^6^ offering two examples of activist networks in Australia. However, it is not enough for (often privileged, rarely with lived experience) academics to do this advocacy alone if these networks are not working with and opening spaces for the amplification of the voices of people with lived experience of forced migration. ‘Caring with’ does not mean speaking for, and we acknowledge that in writing this article as two activist academics we are complicit in these logics. However, we also argue that solidarity can extend to allyship/co-conspiratorship. However, the notion of solidarity, and the language used, are clearly contested; as Carlson et al. (2020) note, we should seek to avoid terminology that ‘simply reproduce[s] an oppressed vs. privileged binary’ (p. 895). The debate about what solidarity can/should look like is unlikely to be resolved quickly, and really requires us to go back to the first of Tronto’s dimensions of an ethics of care (caring about/ attentiveness) to ask and listen to how we can *care with* SfRBs. This is an area of work that is urgent and necessary, if we are to avoid the kinds of representations of/for SfRBs that essentialise refugee experience and become the kinds of ‘inspiration porn’ rightly critiqued by activists like Stella Young (2014), which can be seen in university media portrayals of ‘resilient refugees’ (see also Stevenson & Baker, 2018).

## Conclusions

By focusing on the relational component of our engagement with higher education—as students, educators, researchers, support staff, parents, community members, and advocates—we can develop a better understanding of the moral context of higher education. Applying an ethics of care lens in dialogue with SAMHSA’s 4 Rs for becoming trauma-informed, helps us to remember that we are all deeply imbricated in webs of care and caring. Thinking about university supports as trauma-informed care could help develop more responsive supports to needs, and these are likely to benefit *all* students as a result of assumptions being challenged (Stevenson & Baker, 2018). We are forced to disrupt ‘business-as-usual’ when we purposefully consider the intersecting needs that students like SfRBs have, resulting from likely past trauma and dislocation, and which are exacerbated by subtle cultural differences, gendered roles and barriers to education (Burke et al., 2022), and the impacts of displacement on mental health (Wilson, 2022). These include assumptions about familiarity with education systems, attendance of Australian schools, familiarity with English language and formal literacies, and access to digital infrastructure and equipment.

Relationality is a core tenet of the ethics of care approach, as per the ethos of ubuntu and kinship care that push against western-individualist models. In the contemporary higher education context, characterised by its reliance on the market, competition, and new public managerialism (Blackmore, 2020), our interconnectedness has been diminished, if not lost; as a result, identifying/ considering trauma is more difficult for educators and students to do, especially without support from the institution. This represents a critical challenge to the project of higher education as a public good. As Walker-Gleaves (2019) laments, this translates into a problematic invisibilisation of care in the teacher–student relationship, with the idea of care becoming ‘exceptional’ rather than an integrated part of nurturing students’ holistic development and wellbeing. As such, universities should provide more people-rich resources to support students, so they can build trusting relationships with identified support people, with these roles funded by operational (long term), rather than short-term funding.

Concerns around whose morals are represented and how the discourse on privilege and morals shapes educators who work with refugee communities has prompted the opening of a shared space to theorise, to re-map (some of) the terrain of higher education, especially the response to SfRBs’ transitions into Australian tertiary institutions (Baker & Irwin, 2021; Naidoo, 2015). When read through an ethics of care lens, there is a clear moral conflict at play when universities accept students with specific ‘needs’, such as SfRBs, but do not consult with students as to what they need, nor adjust their practices and structures to accommodate those students. Such p/maternalistic thinking erodes students’ agency in ways that can critically disadvantage their participation and success in their studies. Consequently, we argue that universities be made more accountable for providing support services that meet SfRBs’ needs, with trauma-informed program design developed in close consultation with these students.

However, this should not stop us from pursuing a more caring system that benefits not only SfRBs, but also all students *and* staff. We are morally compelled to rethink our systems, policies, and practices if we view higher education through the twin lenses of ethics of care and SfRBs because we can see all the ways that the academy is failing to care in ways that have significant implications for students, staff, and the institution. It is the ethics of care that contributes to critical consciousness and hence the transformation of the lifeworlds of SfRBs. It is, therefore, imperative that to **realise** (care about)**, recognise** (care for)**, respond** (care give/receive) and **resist** (care with), universities first listen (be attentive) so they can then take responsibility, demonstrate their competence, respond, and show solidarity. Through effective supports using a trauma-informed ethics of care, students can then also learn how to care in ways that are self-determining and for self and others. Without explicit modelling, we cannot assume that this will happen. As the dominant partner in study, universities have a duty to act first and demonstrate their care.

The ethics of care, therefore, is not only about what can be proved, but it is also about connecting the educator to the human condition. However, as educators and researchers, we must also recognise our own limits, and fight to open space for more self-advocacy and empowerment. When it comes to SfRBs, many—including us—have not experienced forced migration and the ways that it impacts on a person. In our case, we write as advocate/activist scholars who engage in research to be attentive, to use our research to be competent and give care through our scholarship *and* our practice (as teacher educators, as advocates, as so-called ‘public intellectuals’). This acknowledgement of our relative privilege could stymy our efforts to care, because of our guilt, shame, and frustration. To avoid this, we follow Zembylas et al. (2014) who argue that an ethics of care requires commensurate attention to critical pedagogies of emotion, to help us to examine our privileged irresponsibility that allows some of us to not care, or to care less because we are distant from the challenges of inadequate care, and to move beyond caregivers’ emotions (anger, shame, guilt, joy) to find positive and productive responses ‘towards relational responsibility and attentiveness’ (p. 208).

In using this trauma-informed ethics of care framework to analyse how universities respond to the needs of marginalised students from refugee backgrounds (SfRBs), we highlight the prevalence of tacit assumptions that inadvertently create under- or uncaring higher education systems. We argue that the one-size-fits-all model is not working and the business-as-usual model that privileges the efficiency of university systems over student needs must be disrupted. To utilise a trauma lens to support SfRB, staff must be trained in trauma awareness and national not piecemeal policies need to be developed to reflect this approach. Accordingly robust funding to support the policies and new practices must be made available. As we process the impacts of COVID and the stark shifts to online teaching and learning, and a refreshed focus on ‘the student experience’, we have called for greater attention to be paid to developing caring and ethical structures that can create more inclusive experiences for SFRB students.

## Footnotes

1 While we use this label throughout as a shorthand to signify the commonalities of forced migration experience, we acknowledge that this cohort are richly heterogeneous, and we do not intend to essentialise identities or experiences.

2 Settlement countries offer permanent residency to refugees.

3 While global mobility was curtailed by the impacts of Covid-19, Australia put its migration program on hold during 20,202,021 and reduced its humanitarian program to 13,750 places. Prior to Covid-19, Australia had increased its humanitarian migration program to 18,750 places. In 2022, a new Labor government reinstated the intake level to 18,750 places.

4 The term ‘traditional’ as used in this article refers to students who are middle class, English-speaking and articulate into university via a school pathway.

5 More information about the RESIG can be found here: www.refugee-education.org

6 More information about Academics for Refugees can be found here: https://academicsforrefugees.wordpress.com

## References

[CR2] Bajwa J, Couto S, Kidd S, Markoulakis R, Abai M, McKenzie K (2017). Refugees, higher education, and informational barriers. Refuge: Canada’s Journal on Refugees.

[CR3] Baker S, Due C, Rose M (2021). Transitions from education to employment for culturally and linguistically diverse migrants and refugees in settlement contexts: What do we know?. Studies in Continuing Education.

[CR4] Baker S, Irwin E (2021). Disrupting the dominance of ‘linear pathways’: How institutional assumptions create ‘stuck places’ for refugee students’ transitions into higher education. Research Papers in Education.

[CR5] Baker S, Irwin E, Freeman H (2020). Wasted, Manipulated and Compressed Time: Adult Refugee Students’ Experiences of Transitioning into Australian Higher Education. Journal of Further and Higher Education.

[CR6] Baker S, Ramsay G, Irwin E, Miles L (2018). ‘Hot’, ‘Cold’ and ‘Warm’ Supports: Towards theorising where refugee students go for assistance at university. Teaching in Higher Education.

[CR7] BarnacleDall’Alba RG (2017). Committed to learn: Student engagement and care in higher education. Higher Education Research & Development.

[CR8] Berger, E., & Martin, K. (2020, Sep 11). *Why every teacher needs to know about childhood trauma*. The Conversation. Retrieved February 10, 2023, from https://theconversation.com/why-every-teacher-needs-to-know-about-childhood-trauma-132965?gclid=CjwKCAiA5sieBhBnEiwAR9oh2iikVmPyJHdeDONxzIn2j4LV5DFdk2zU0yZ5IOLohIaW5HLEUEImtxoC8sIQAvD_BwE

[CR9] Blackmore J (2020). The carelessness of entrepreneurial universities in a world risk society: A feminist reflection on the impact of Covid-19 in Australia. Higher Education Research & Development.

[CR10] Bosanquet, A. (2017). Undercare in the academy. *The Slow Academic.* Retrieved from https://theslowacademic.com/?s=undercare

[CR11] Bozalek V, McMillan W, Marshall D, November M, Daniels A, Sylvester T (2014). Analysing the professional development of teaching and learning from a political ethics of care perspective. Teaching in Higher Education.

[CR12] Burke R, Baker S, Hartley L, Field R (2022). What do we know about how women with forced migration experiences access tertiary education in resettlement contexts?. A Scoping Study. Gender and Education. Retrieved from:.

[CR13] Carlson J, Leek C, Casey E, Tolman R, Allen C (2020). What’s in a name? A synthesis of ‘allyship’ elements from academic and activist literature. Journal of Family Violence.

[CR15] Fisher B, Tronto J, Abel E, Nelson M (1990). Towards a feminist theory of caring. Circles of Care: Work and Identity in Women’s Lives.

[CR16] Grummell B, Devine D, Lynch K (2009). The care-less manager: Gender, care and new managerialism in higher education. Gender and Education.

[CR17] Halkic B, Arnold P (2020). Refugees and online education: Student perspectives on need and support in the context of (online) higher education. Learning, Media and Technology.

[CR18] Hegney D, Tsai L, Craigie M, Crawford C, Jay S, Rees C (2021). Experiences of university employees of the impact of a mindful self-care and resiliency program on their well-being. Higher Education Research & Development.

[CR19] Lambrechts A (2020). The super-disadvantaged in higher education: Barriers to access for refugee background students in England. Higher Education.

[CR20] Mangan D, Winter L (2017). (In)validation and (mis)recognition in higher education: The experiences of students from refugee backgrounds. International Journal of Lifelong Education.

[CR21] Marcu S (2018). Refugee students in Spain: The role of universities as sustainable actors in institutional integration. Sustainability.

[CR22] Mariskind C (2014). Teachers’ care in higher education: Contesting gendered constructions. Gender and Education.

[CR23] Matthews N, Sunderland N (2017). Digital storytelling in health and social policy: listening to marginalised voices.

[CR24] McDowell L (1992). Doing gender: Feminism, feminists and research methods in human geography. Transactions, Institute of British Geographers.

[CR25] McWilliams J, Bonet S (2016). Continuums of precarity: Refugee youth transitions in American high schools. International Journal of Lifelong Education.

[CR26] Molla T (2022). African refugee youth: Higher education participation. Higher Education Research and Development.

[CR27] Mupenzi A, Mude W, Baker S (2020). Reflections on COVID-19 and impacts on equitable participation: The case of culturally and linguistically diverse migrant and/ or refugee (CALDM/R) students in Australian higher education. Higher Education Research & Development.

[CR28] Naidoo L (2015). Educating refugee-background students in Australian schools and universities. Intercultural Education.

[CR29] Naidoo L, Wilkinson J, Adoniou M, Langat K (2018). Navigating the Terrain of Higher Education. Refugee background students transitioning into higher education.

[CR30] Noddings N (1984). Caring: A feminine approach to ethics and moral education.

[CR31] Ollerhead S, Baker S, Johnson H, Anderson V (2019). Is there any appetite for ‘linguistic hospitality’ in monolingual educational spaces? The case for translanguaging in Australian higher education. Migration, Education & Translation.

[CR32] Perales, F., Kubler, M., Xiang, N. & Tomaszewski, W. (2021). *Understanding access to higher education amongst humanitarian migrants in Australia*. National Centre for Student Equity in Higher Education. Retrieved from https://www.ncsehe.edu.au/wp-content/uploads/2021/03/Perales_UQ_HumanitarianMigrants_FINAL.pdf

[CR33] Stephens DW (2020). Trauma-Informed Pedagogy for the religious and theological higher education classroom. Religions.

[CR34] Stevenson, J., & Baker, S. (2018). Refugees in higher education: Debate, discourse and practice. Emerald Group Publishing.

[CR35] Streitweiser B, Brück L (2018). Competing motivations in Germany’s higher education response to the ‘refugee crisis’. Refuge: Canada’s Journal on Refugees.

[CR36] Substance Abuse and Mental Health Services Administration. (2014). *SAMHSA’s concept of trauma and guidance for a trauma-informed approach* (HHS Publication No. (SMA) 14−4884. Rockville, MD.

[CR37] Swartz BC, Gachago D, Belford C (2018). To care or not to care reflections on the ethics of blended learning in times of disruption. South African Journal of Higher Education.

[CR38] Tronto J (1993). Moral boundaries: A political argument for an ethic of care.

[CR39] Tronto J (2013). Caring democracy: Markets, equality, and justice.

[CR40] Van Der Kolk B (2014). The Body Keeps The Score.

[CR41] Vásquez Vedera V (2019). Care ethics in universities: Beyond an easy ‘add and stir’ solution. Encounters in Theory and History of Education.

[CR42] Waghid Y (2019). Towards a Philosophy of Caring in Higher Education Pedagogy and Nuances of Care.

[CR43] Walker C, Gleaves A (2016). Constructing the caring higher education teacher: A theoretical framework. Teaching and Teacher Education.

[CR44] Walker-Gleaves C, Gibbs P, Peterson A (2019). Is caring pedagogy really so progressive? Exploring the conceptual and practical impediments to operationalizing care in higher education. Higher Education and Hope.

[CR45] Warin J (2014). The status of care: Linking gender and ‘educare’. Journal of Gender Studies.

[CR46] Wilson V. (2022) Liberty, Equality, Fraternity: Trauma-Informed English Language Teaching to Adults. The Paris Conference on Education 2022: Official Conference Proceedings*.*10.22492/issn.2758-0962.2022.19

[CR47] Young, S. (2014). *I'm not your inspiration, thank you very much* [Video]. TED Conferences. https://www.ted.com/talks/stella_young_i_m_not_your_inspiration_thank_you_very_much/transcript?language=en

[CR48] Zembylas M, Bozalek V, Shefer T (2014). Tronto’s notion of privileged irresponsibility and the reconceptualisation of care: Implications for critical pedagogies of emotion in higher education. Gender and Education.

